# FUSI-CAD: Coronavirus (COVID-19) diagnosis based on the fusion of CNNs and handcrafted features

**DOI:** 10.7717/peerj-cs.306

**Published:** 2020-10-12

**Authors:** Dina A. Ragab, Omneya Attallah

**Affiliations:** Electronics and Communications Engineering Department, Arab Academy for Science, Technology, and Maritime Transport (AASTMT), Alexandria, Egypt

**Keywords:** Computer-aided diagnosis (CAD), Convolution neural networks (CNN), Coronavirus (COVID-19), Discrete wavelet transform (DWT), Grey level co-occurrence matrix (GLCM)

## Abstract

The precise and rapid diagnosis of coronavirus (COVID-19) at the very primary stage helps doctors to manage patients in high workload conditions. In addition, it prevents the spread of this pandemic virus. Computer-aided diagnosis (CAD) based on artificial intelligence (AI) techniques can be used to distinguish between COVID-19 and non-COVID-19 from the computed tomography (CT) imaging. Furthermore, the CAD systems are capable of delivering an accurate faster COVID-19 diagnosis, which consequently saves time for the disease control and provides an efficient diagnosis compared to laboratory tests. In this study, a novel CAD system called FUSI-CAD based on AI techniques is proposed. Almost all the methods in the literature are based on individual convolutional neural networks (CNN). Consequently, the FUSI-CAD system is based on the fusion of multiple different CNN architectures with three handcrafted features including statistical features and textural analysis features such as discrete wavelet transform (DWT), and the grey level co-occurrence matrix (GLCM) which were not previously utilized in coronavirus diagnosis. The SARS-CoV-2 CT-scan dataset is used to test the performance of the proposed FUSI-CAD. The results show that the proposed system could accurately differentiate between COVID-19 and non-COVID-19 images, as the accuracy achieved is 99%. Additionally, the system proved to be reliable as well. This is because the sensitivity, specificity, and precision attained to 99%. In addition, the diagnostics odds ratio (DOR) is ≥ 100. Furthermore, the results are compared with recent related studies based on the same dataset. The comparison verifies the competence of the proposed FUSI-CAD over the other related CAD systems. Thus, the novel FUSI-CAD system can be employed in real diagnostic scenarios for achieving accurate testing for COVID-19 and avoiding human misdiagnosis that might exist due to human fatigue. It can also reduce the time and exertion made by the radiologists during the examination process.

## Introduction

The novel virus known as the severe acute respiratory syndrome coronavirus 2 (SARS-CoV-2) has emerged in December 2019 in Wuhan, China. Patients diseased with SARS-CoV-2 can experience a medical condition known as coronavirus diseases 2019 (COVID-19). This new coronavirus has quickly spread to other zones in China and other countries with significant morbidity and mortality rates. On April 20, 2020, more than 84,000 COVID-19 infected patients have been verified in China, and more than 2.3 million patients worldwide (Dong et al., 2020). COVID-19 may lead to critical respiratory illness, acute pneumonia, numerous organs failure, and even death (Cheng et al., 2020). For this reason, the early detection of this virus is essential, as it is achieving a primary initial assessment so that the succeeding arrangement of suitable treatments and follow-ups can be set in advance. Pandemic COVID-19 has led to a rapid increase in the number of new patients. Consequently, an overload in hospital capacity occurred, and healthcare management became a difficult task (Wu et al., 2020; Paules, Marston & Fauci, 2020). In addition, the doctors and nurses are at risk of being infected, and thus they will not be capable of helping patients and managing their jobs (Butt et al., 2020). Therefore, a rapid and accurate diagnostic tool is crucial to help doctors in managing their patients.

The standard way of screening infected patients is known as the reverse-transcription polymerase chain reaction (RT-PCR) testing (Dong et al., 2020). Although RT-PCR is the common method to diagnose COVID-19 cases, it has some limitations. First, the sensitivity of the RT-PCR test is not quite high, and therefore COVID-19 disease may not be completely omitted, despite if RT-PCR outcomes from a suspected case are negative (Ai et al., 2020). Moreover, the inadequate quantity and firm necessities for laboratory settings would critically postpone precise identification of suspected individuals, which has modeled extraordinary challenges to avoid the propagation of the disease worldwide, specifically in the middle of the epidemic region (Xie et al., 2020). Thus, medical imaging, in specific, chest-computed tomography (CT), is preferred as another tool in the diagnosis and management of the novel coronavirus. CT offers a faster and simpler solution for medical diagnosis of COVID-19. It is used as well in observing of disease progression and the assessment of treatment efficiency (Iwasawa et al., 2020; Attallah et al., 2017a).

CT is considered as the main element of the diagnostic and treatment process for suspected cases due to its manifestations, which have been reported in several recent articles (Lei et al., 2020; Chung et al., 2020; Song et al., 2020a). Moreover, it can be used alone by the radiologists due to the overlap of the appearance of COVID-19 with other types of pneumonia, which challenges the examination process (Shi, Han & Zheng, 2020). Furthermore, manual examination using CT images needs lots of manual employment time-consuming. Therefore, the health industry is considering other new technologies to screen and control the spread of the novel coronavirus pandemic. Artificial intelligence (AI) is an example of such technologies that can tackle the propagation of such disease, recognize patients at risk, control this virus in real-time, and automatically detect and diagnose suspected cases (Vaishya et al., 2020). Machine learning (ML) and deep learning (DL) are classes of AI, which have the potential to enhance the diagnosis and treatment planning of COVID-19 cases, being an evidence-based medical tool (Attallah et al., 2017b, 2017a; Karthikesalingam et al., 2015).

Computer-aided diagnosis (CAD) systems based on AI techniques such as ML and specifically DL have been described as an effective tool to diagnose and detect numerous diseases and abnormalities from medical images (Ragab, Sharkas & Attallah, 2019; Attallah, Sharkas & Gadelkarim, 2019; Ragab et al., 2019; Attallah, Sharkas & Gadelkarim, 2020; Attallah, Gadelkarim & Sharkas, 2018). The CAD systems were used to diagnose several lung diseases such as tuberculosis (Ke et al., 2019). The authors in Wei et al. (2019b) and Capizzi et al. (2019) used CT images to classify lung nodule images. Recently, the CAD systems have been used for diagnosing and identifying COVID-19 disease from other types of pneumonia. The authors in Wieczorek, Silka & Woźniak (2020) employed a multicenter study that combines CT images for COVID-19 patients from several countries to deliver probably a wide range of values about predicted COVID-19 spread. The authors constructed a convolutional neural network (CNN) architecture, which is consists of seven layers. The network is trained by NAdam, which is selected in tests for the greatest effectiveness and shortest training time achieving an accuracy 99%. Zheng et al. (2020) used UNet++ to segment lesions in CT images. Afterward, the bounding box of the segmented lesion was generated. Finally, a 3D-CNN was constructed for predicting the probability of COVID-19. The proposed method reached a sensitivity of 90.7%, a specificity of 91.1%, and an area under the receiver-operating curve (AUC) of 0.959 (95.9%). There were two limitations in this technique; first, the number of cases was small. Moreover, the UNet++ used in the segmentation of images may result in segmenting the infection areas that have small blood vessels that reduce the performance of the CAD system. The authors in Fang et al. (2020) applied radiomics analysis from a manually delineated region of interest (ROI) CT images to diagnose COVID-19. Afterward, unsupervised consensus clustering was used to choose significant features. Finally, a support vector machine (SVM) classifier was used to classify COVID-19. This study achieved an AUC of 0.826 (82.6%). The benefit of this study is using radiomics quantitative analysis as a feature extractor, which is considered as a powerful extractor along several medical domains (Lambin et al., 2012; Kolossváry et al., 2017). However, the main drawback of Fang et al. (2020) method is that the authors used only handcrafted features and discarded the advantages of DL techniques, and therefore, they did not attain high performance. Li et al. (2020) proposed a technique that depends on ResNet-50 CNN to classify COVID-19. The sensitivity, specificity, and AUC produced were 87%, 92%, and 0.95 (95%), respectively. The privilege of such a technique is that it used large amount of images. Moreover, it utilized a heatmap to picture the important areas in the images in the classification. Nevertheless, the heatmaps are not yet sufficient to capture what distinctive features are utilized by the model to differentiate between COVID-19 and non-COVID-19.

Furthermore, Ardakani et al. (2020) compare the performance of 10 popular CNN to differentiate COVID-19 from non-COVID-19 patients. The results showed that ResNet-101 and Xception CNNs have the highest performance. The accuracy, AUC, sensitivity, and specificity obtained by ResNet-101 CNN were 99.51%, 0.994 (99.4%), 100%, and 99.02%, respectively. However, the Xception CNN attained an accuracy of 99.02%, an AUC of 0.994 (99.4%), a sensitivity of 98.04%, and a specificity of 100%. The main advantages of Ardakani et al. (2020) method are using very high-resolution images and splitting each image in the dataset into several patches that are then used to train the CNN. The authors in Singh, Kumar & Kaur (2020) introduced a novel CNN and tuned its initial parameters by using a multi-objective differential evolution (DE) method, which was the key privilege of their CAD system. The DE algorithm has confirmed its effectiveness in various domains (Zhang & Sanderson, 2009). This evolution technique guarantees that the individual that has superior merits is left as a portion of the population and fragile individuals are detached with each iteration (Hancer, Xue & Zhang, 2018). The results obtained were almost 93%. However, the limitation of Singh, Kumar & Kaur (2020) and Ardakani et al. (2020) study is they did not compare the performance of their CAD system with that of the radiologist. He et al. (2020) employed several CNNs to classify COVID-19 cases. The best performance was achieved by DenseNet-169 where, the accuracy and AUC were 86% and 0.94 (94%), respectively. The DenseNet is a novel CNN architecture, which can perform well in case of trained with a huge number of images; however, it has a high complexity and a large number of layers, which increase the chances of overfitting in case of trained with inadequate number of images. Hasan et al. (2020) proposed a hybrid approach based on CNN, Q-deformed entropy, and long-short-term-memory (LSTM) network and accomplished an accuracy of 99.68%. The advantage of this method is that the authors constructed a new CNN with few number of convolutional layers to decrease the over-fitting by reducing the CNN construction complexity. They also utilized a new feature extractor called Q-deformed entropy, which is a textural analysis method capable of providing a major benefit in medical image classification. In addition, the Q-deformed entropy is an entropy based technique that can detect small alterations in the image’s intensity values, along with sharpening the texture details (Al-Shamasneh et al., 2018). The authors in Bai et al. (2020) employed EfficientNet-B4 to identify COVID-19 with an accuracy of 87%, AUC of 0.9 (90%), sensitivity of 89%, and a specificity of 86%. The study of Bai et al. (2020) has compared the performance of the CAD system based on AI techniques with manual diagnosis by radiologists. It proved that the AI assistance had enhanced the radiologists’ performance in diagnosing COVID-19 cases, but there could be a bias as a result of the radiologist’s evaluation of the same suspects twice, primary without, and then with AI assistance, whereas the authors in Amyar, Modzelewski & Ruan (2020) used two U-Nets to diagnose COVID-19. The first network was used to segment the lung from the rest of the image, while the other one for classification. The accuracy, AUC, sensitivity, and specificity achieved were 86%, 0.93 (93%), 94%, and 79%, respectively. The benefit of this method is using a multi-task learning method, which fuses several portions of information from different tasks to enhance the performance of the CAD system and its capability to better generalize. U-Net was used as well in Chen et al. (2020) to discriminate COVID-19 and non-COVID-19. The results obtained showed an accuracy of 95.2%, a sensitivity of 100%, and a specificity of 93.6%. The reason of such good results is the huge number of images used to train their U-NETs.

A summary of similar studies is shown in Table 1. As it is obvious from the table that most of the related work is based on DL techniques, which either employing a single CNN or combining two CNNs where the first CNN is used for segmentation and the other is for the classification and diagnosis of COVID-19. However, the process of fusing multiple CNNs is not well examined in previous studies. Moreover, almost all of the existing studies depend on DL only as a feature extraction, which may have some limitations regarding feature extraction complexity. Feature extraction is a vital process for extracting important spatial variations of the image pixels. Although, DL is considered a powerful tool for large-scale image classification and feature extraction, however, it could not be the ideal choice when the dataset available is not large containing a small number of images (Nguyen et al., 2018). DL techniques may hardly employ heuristics to direct feature extraction for every particular task because of the automatic feature extraction procedure. They can also face the problem of overfitting when the training dataset is not large (Zhang et al., 2017; Nanni, Ghidoni & Brahnam, 2017). Fusing DL and handcrafted features may improve the performance of the image classification problems (Hasan et al., 2020; Wei et al., 2019a; Hansley, Segundo & Sarkar, 2018). Therefore, in this study, we proposed an efficient novel CAD system called FUSI-CAD, which investigates the effect of fusing handcrafted features and DL features extracted from multiple CNNs trained with COVID-19 CT images. The proposed system as it will be discussed later consists of three stages:

- Stage (1)—Deep features fusion: This is performed by extracting and fusing the deep features of the fine-tuned AlexNet, GoogleNet, ShuffleNet, and ResNet-18 CNN architectures.
- Stage (2)—Handcrafted features fusion: This is accomplished by extracting and fusing three types of handcrafted (HC) features; the statistical features, discrete wavelet transform (DWT), and grey level co-occurrence matrix (GLCM) features.
- Stage (3)—FUSI-CAD (DL and HC feature fusion): This is implemented by fusing the features of both stages (1) and (2).

The FUSI-CAD system compares its performance with stages (1) and (2) to test the effect of fusing multiple DL features with three HC features on the performance of the CAD system.

The novelty of the FUSI-CAD system is concentrated in several contributions:

- Exploring various CNN based approaches different than that have been utilized in the literature for detecting the presence of COVID-19 from chest CT images.
- Fusing features extracted from the deep layers of CNNs to diagnose COVID-19 patients, as the fusion of deep features was not examined in the literature.
- Combining powerful handcrafted features to diagnose COVID-19 patients, as this was not examined in the literature.
- Comparing the usage of deep features with handcrafted features for diagnosing COVID-19 with a dataset of few numbers of images.
- A feature fusion based approach is proposed, which combines deep features from multiple individual CNNs models with three powerful handcrafted features based on textural analyses, to produce a final accurate and efficient diagnostic tool even with a COVID-19 dataset containing small number of images.
- Developing a final fused model (multiple deep features and three handcrafted features), which is able to verify that each of the two feature extraction paradigms is capable of extracting information that is neglected by the other paradigm.
- Constructing a reliable model that is faster and more accurate than the conventionally used RT-PCR testing kit.

**Table 1:** A summary of recent similar related studies.

Paper	Dataset	Method	Results
Butt et al. (2020)	219 COVID-19 339 Others	ResNet-18 and ResNet-23	Accuracy = 95.2% AUC = 0.996 (99.6%) Sensitivity = 98.2% Specificity = 92.2%
Li et al. (2020)	468 COVID-19 2,996 Others	ResNet-50	Accuracy = 89.5% AUC = 0.95 (95%) Sensitivity = 87% Specificity = 92%
Bai et al. (2020)	521 COVID-19 665 others	EfficientNet-B4	Accuracy = 87% AUC = 0.9 (90%) Sensitivity = 89% Specificity = 86%
He et al. (2020)	191 COVID-19 234 others	DenseNet-169	Accuracy = 86% AUC = 0.94 (94%)
Amyar, Modzelewski & Ruan (2020)	449 COVID-19 595 others	Two U-Nets	Accuracy = 86% AUC = 0.93 (93%) Sensitivity = 94% Specificity = 79%
Ardakani et al. (2020)	108 COVID-19 86 others	ResNet-101	Accuracy = 99.51% AUC = 0.994 (99.4%) Sensitivity = 100% Specificity = 99.02%
		Xception	Accuracy = 99.02% AUC = 0.994 (99.4%) Sensitivity = 98.04% Specificity =100%
Chen et al. (2020)	4,382 COVID-19 9,369 others	U-Net++	Accuracy = 95.2% Sensitivity = 100% Specificity = 93.6%
Zheng et al. (2020)	313 COVID-19 229 Others	U-Net and CNN	Accuracy = 90.9% AUC = 0.959 (95.9%) Sensitivity = 90.7% Specificity = 91.1%
Jin et al. (2020b)	723 COVID-19 413 Others	U-Net and ResNet-50	Accuracy = 94.8% Sensitivity = 97.4% Specificity = 92.2%
Hasan et al. (2020)	118 COVID-19 203 Others	CNN,Q-deformed entropy, and LSTM	Accuracy = 99.68%
Jin et al. (2020a)	496 COVID-19 1,385 Others	Deeplab-v1 and ResNet 152	Accuracy = 94.8% AUC = 0.979 (97.9%) Sensitivity = 94.1% Specificity = 95.5%
Song et al. (2020b)	219 COVID-19 399 Others	ResNet-50	Accuracy = 86% AUC = 0.95 (95%) Sensitivity = 96% Precision = 79%
Singh, Kumar & Kaur (2020)	–	CNN	Accuracy~93%
Wang et al. (2020)	325 COVID-19 740 Others	Inception and Adaboosted decision tree	Accuracy = 82.9% AUC = 0.9 (90%) Sensitivity = 81% Specificity = 84%
Fang et al. (2020)	46 COVID-19, 29 others	Radiomic feature extraction, clustering and SVM	AUC = 0.826 (82.6%)

## Methods and Materials

### Dataset description

**Figure 1:**
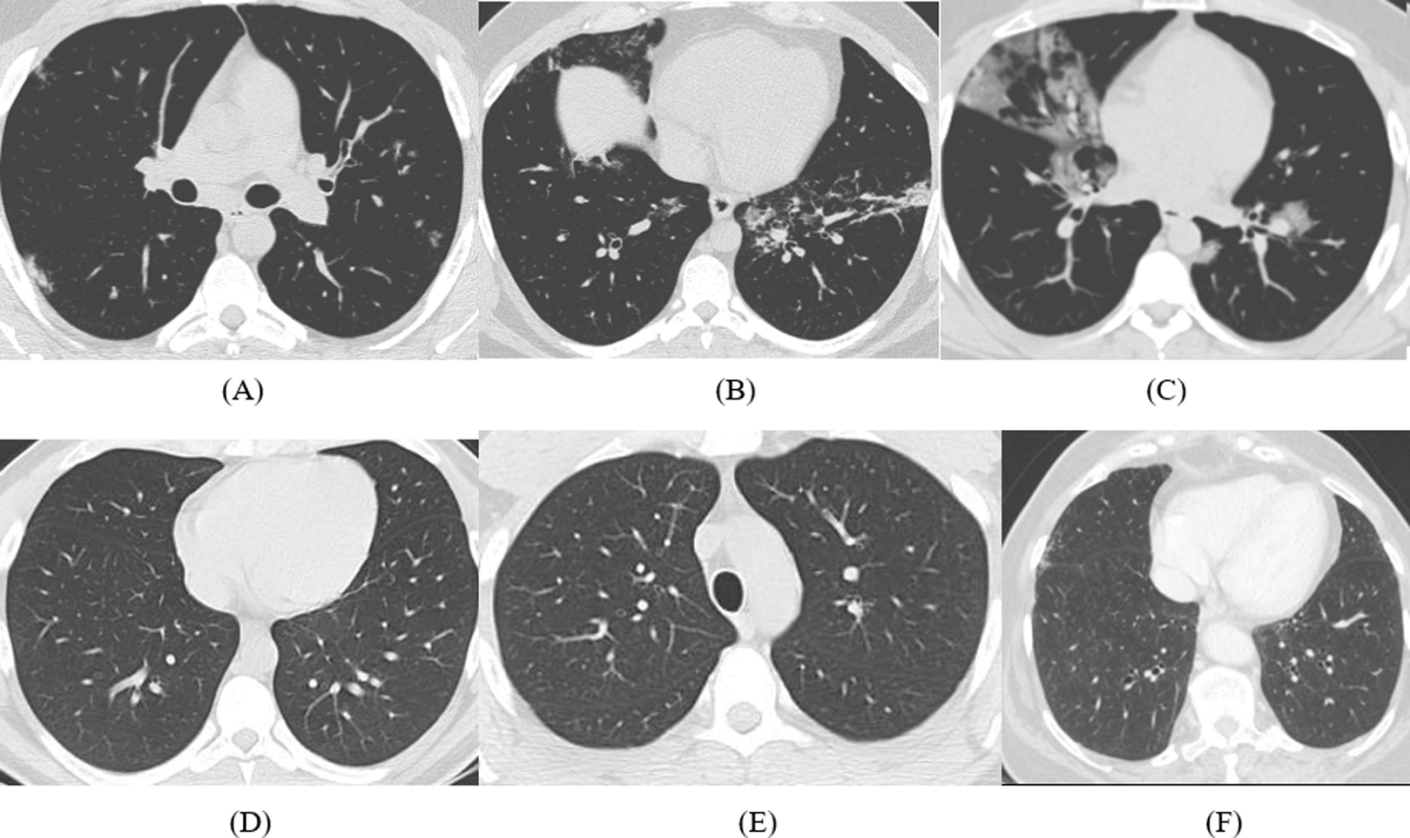
Samples of CT images (A)–(C) COVID-19 CT images and (D)–(F) non-COVID-19 CT images.

The dataset used is SARS-CoV-2 CT-scan dataset. It is acquired from a hospital in Sao Paulo, Brazil, and permitted by their ethical committee. The description of this dataset is available in Soares et al. (2020). The dataset contains 1252 CT-images of COVID-19 positive cases for 60 patients (32 male +28 female) and 1230 CT-Scans for non-COVID-19 cases for 60 normal patients (30 male +30 female), which are COVID-19 negative but may have other pulmonary illnesses. Figure 1 shows some samples extracted from the dataset representing CT images for COVID-19 and non-COVID-19.

### Convolutional neural networks

**Figure 2:**
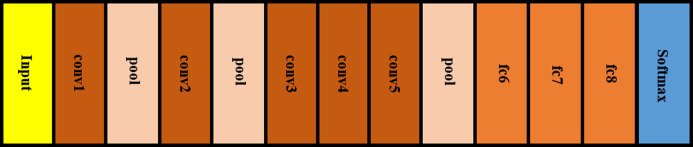
AlexNet CNN architecture.

**Figure 3:**
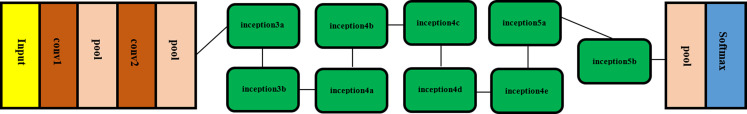
GoogleNet CNN architecture.

**Figure 4:**
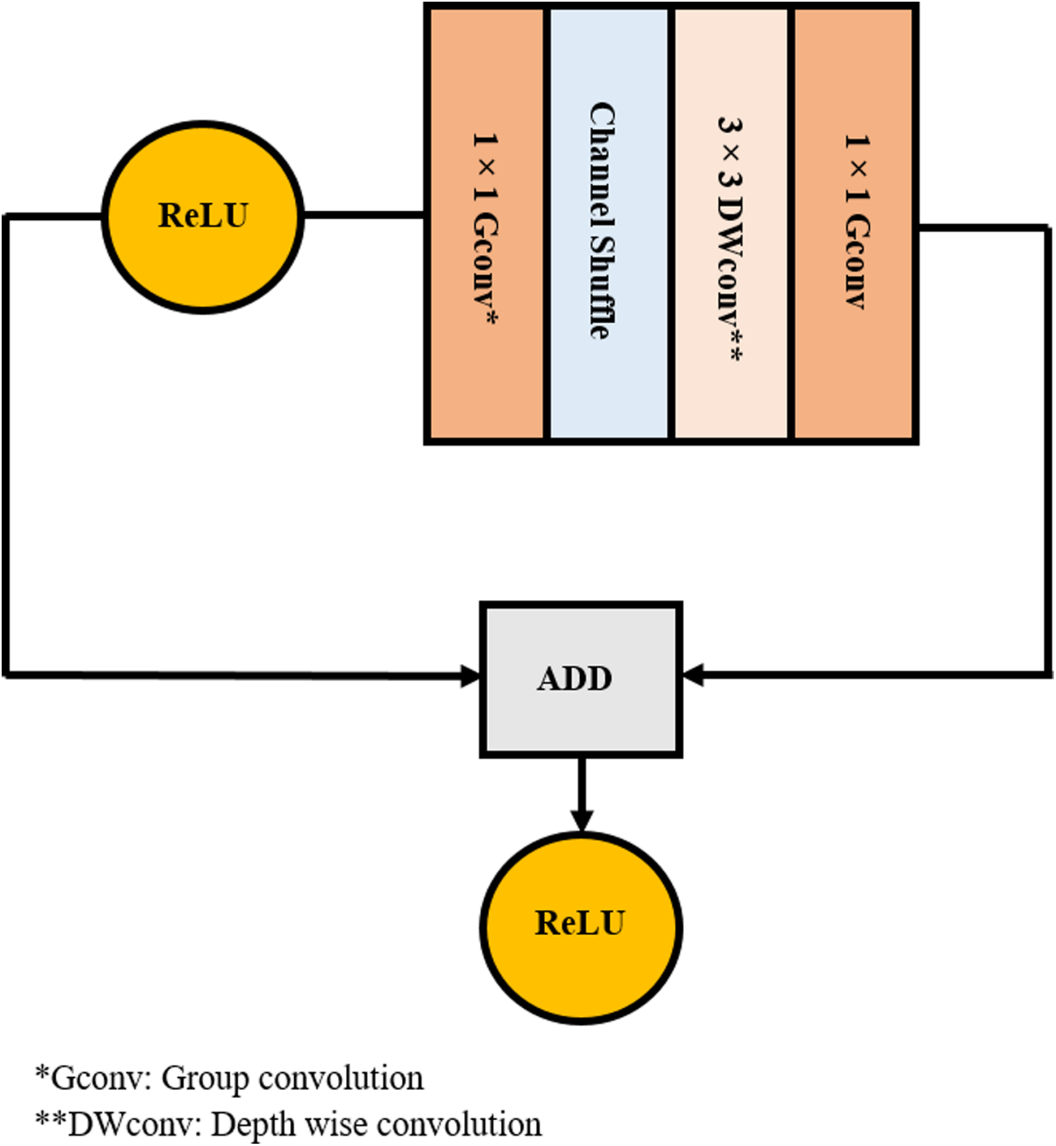
ShuffleNet CNN architecture.

**Figure 5:**
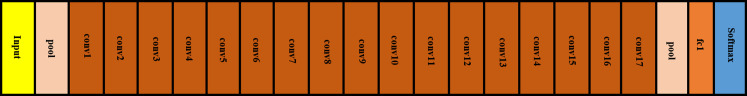
ResNet*-18* CNN architecture.

Convolutional neural network (CNN) is a branch of DL methods that are broadly used for resolving classification problems of medical images in health informatics field (Jin et al., 2020b; Ravì et al., 2016). For this reason, four CNN architectures are employed in this article. Any CNN involves several layers including; convolutional layers, pooling layers, and fully connected (fc) layers. In the first layer, several filters are convolved with the region of the input image corresponding to the same dimension of the filter. Next, a feature map is created representing the position of the features in the original image. Such features characterize the spatial information of the pixel values of the original input image. Then, the pooling layer lowers the huge dimension of the feature map using a down sampling process. Lastly, the fc layer accomplishes the classification procedure similar to the conventional artificial neural network (ANN). CNN can be either used as a classifier or feature extractor. In this paper, four CNNs are employed as feature extractors. Such networks include AlexNet (Krizhevsky, Sutskever & Hinton, 2012) and (Dirvanauskas et al., 2019), GoogleNet (Szegedy et al., 2015), ShuffleNet (Girshick et al., 2014) and ResNet-18 (He et al., 2016) constructions. The architectures of AlexNet, GoogleNet, ShuffleNet, and ResNet-18 CNN are illustrated in Figs. 2–5, respectively. The CNN networks are trained using the ImageNet dataset, which has 1.2 million natural images in 1,000-labelled classes. The transfer learning technique is performed on these networks so that it can be used in any classification problem. This is implemented by replacing the last fully connected layer in any network by a new layer for the classification of two classes: COVID-19 and non-COVID-19. To our knowledge and according to the literature these networks have not yet been employed as feature extractors to classify COVID-19. A summary of the architecture of the four networks is shown in Table 2.

**Table 2:** A summary of the architecture of the four pre-trained CNNs used.

CNN architecture	Number of layers	Input size	Output size
AlexNet	8	227 × 227	4,096 × 2
GoogleNet	22	224 × 224	1,024 × 2
ShuffleNet	50	224 × 224	544 × 2
ResNet-18	18	224 × 224	512 × 2

### The proposed computer-aided diagnosis system

The proposed CAD system consists of four steps, which are image enhancement, feature extraction, feature fusion, and feature classifications steps. In the image enhancement step, images were enhanced using an adaptive contrast enhancement method. Afterward, in the feature extraction step, two types of features were extracted. First, four pre-trained CNNs were constructed and used as feature extractors. These CNNs include AlexNet, GoogleNet, ShuffleNet, and ResNet-18 architectures. Second, three handcrafted (HC) feature extraction methods were employed to extract the features from the CT images. These handcrafted methods consist of statistical features and the textural analysis features; such as discrete wavelet transform (DWT) and grey level co-occurrence matrix (GLCM). Next, the feature fusion step, where features were fused in three stages;

- Stage (1) is the deep features fusion: In this stage, the deep features extracted from the four fine-tuned pre-trained CNN were fused. The influence of DL feature fusion was not examined in the diagnosis of COVID-19, therefore, the novel part is investigating the impact of DL fusion on identifying COVID-19 patients.
- Stage (2) is the handcrafted features fusion: This stage carried out a fusion from three HC feature extractors as mentioned previously. These features were not studied in the related work for coronavirus diagnosis and classification. Therefore, the novel part is using the HC features in coronavirus diagnosis and investigating the effect of combining these three features on the classification results.
- Stage (3) is the FUSI-CAD system: This stage was implemented by fusing the features of both the multiple DL features of stage (1) and the fused handcrafted features of stage (2). The FUSI-CAD system was conducted to verify that fusing the features of stages (1) and (2) can improve the diagnosis performance of COVID-19. To the best of our knowledge, the fusion of DL and HC features was not examined in related CAD system discussing coronavirus diagnosis.

**Figure 6:**
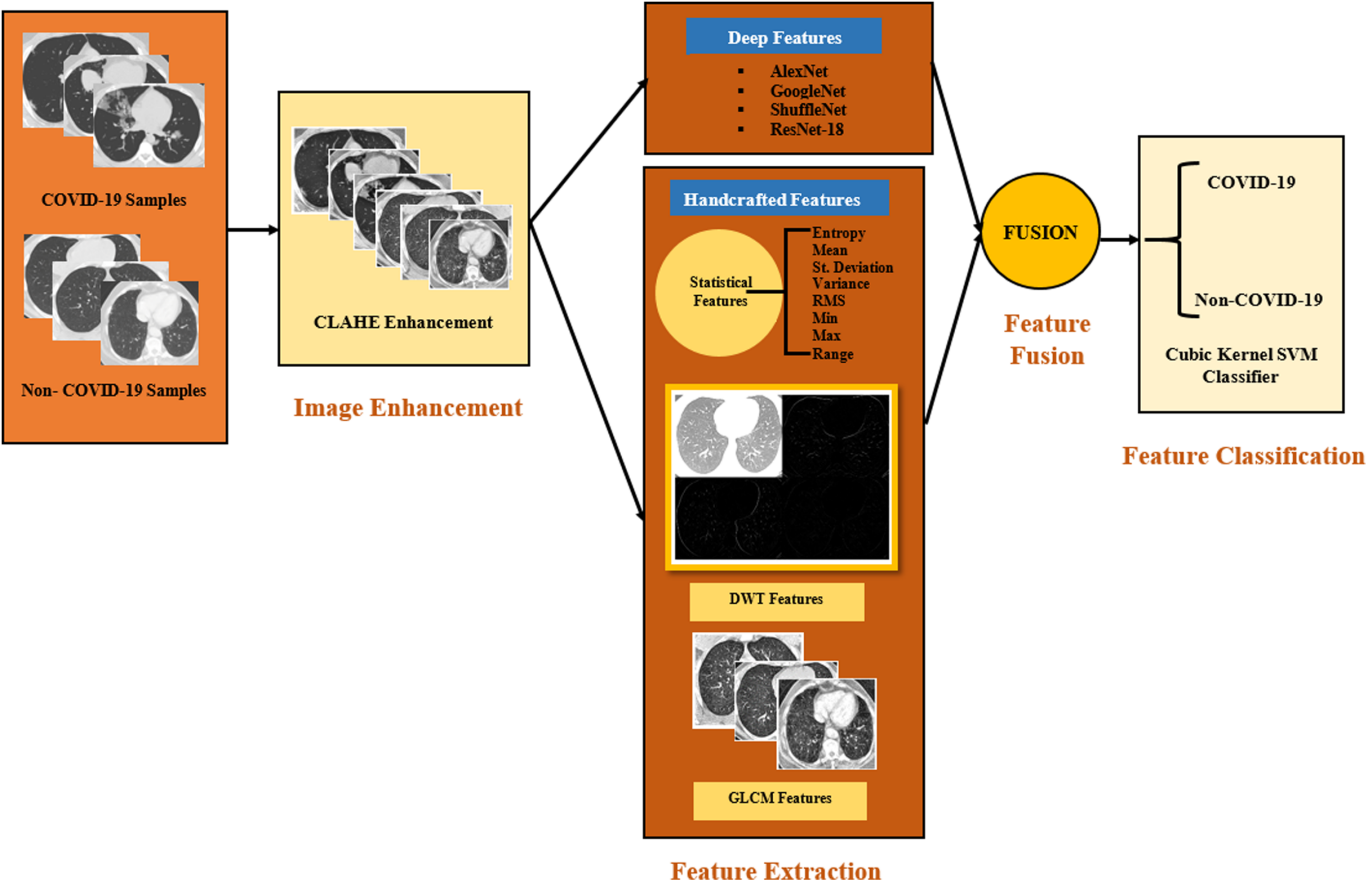
The framework of the proposed CAD system.

Finally, in the classification step, the fused features were used to construct classification models using a support vector machine (SVM) with cubic kernel function to classify CT images into COVID-19 or non-COVID-19. The framework of the proposed CAD system is shown in Fig. 6. The steps of the proposed CAD system is discussed in detail in the following sub-sections.

#### Image enhancement step

Image enhancement is an important process to improve the image quality, contrast, and remove noise to help radiologists in identifying the abnormalities. There are several image enhancement methods; between them is the adaptive contrast enhancement method (AHE). AHE method has the ability to improve the local contrast and express more details in the image. In this study, the contrast-limited adaptive histogram equalization technique (CLAHE); a subclass of AHE is used to enhance the contrast of images (M.Pizer et al., 1987; Pisano et al., 1998). The CLAHE algorithm can be summarized as follows; (Sahakyan, 2012)

1. Split the original CT image into contextual areas,
2. Find a local histogram for each pixel,
3. Bound this histogram established on the clip level,
4. Reorganize the histogram using binary examination, and
5. Get the enhanced pixel value by histogram integration.

#### Feature extraction step

In this step, handcrafted features along with DL features are extracted. The details of the feature extraction step are illustrated in this subsection.

##### Handcrafted feature extraction

Texture analysis feature extraction methods are well-known techniques for mining features from medical images (Castellano et al., 2004; Kassner & Thornhill, 2010; Lahmiri & Boukadoum, 2013; Depeursinge, Al-Kadi & Ross Mitchell, 2017). Such methods depend on the textural characteristics of the image. Textural features extraction methods include the discrete wavelet transform (DWT), and the gray-level co-occurrence matrix (GLCM). These techniques usually yield reasonable classification accuracy especially when they are combined (Nailon, 2010; Lahmiri & Boukadoum, 2013). For this reason, they are used in this study to extract several subsets of features along with statistical features from the spatial domain.

###### Statistical features

In this step, each image was divided into blocks of size 32 × 32. Afterward, eight statistical features were calculated from each block of an image in the spatial domain. Next, these features from all blocks were combined in one feature vector. These features include entropy, mean, standard deviation, variance, root mean square (RMS), minimum, maximum, and range as defined from Eqs. (1)–(6). The size of the statistical features extracted was 512.

(1) $${\rm Entropy} = \; - \mathop \sum \limits_{i = 0}^{n - 1} p{r_{i\; }} \times \log p{r_i}$$

where *n* is the number of grey levels. $p{r_{i\; }}$ is the probability of a pixel having gray level *i*.

(2) $${\rm Mean}({{\rm \mu} _z}) = \displaystyle{1 \over {FG}}\mathop \sum \limits_{i.j = 1}^{FG} {p_z}\left( {i.j} \right)$$

where *p*_z_ (*i*, *j*) is the pixels value in the image block *z*, *F* × *G* is the size of each block *z*.

(3) $${\rm Standard\; deviation}\ \left( {{{\rm \sigma} _z}} \right) = \; \sqrt {\displaystyle{1 \over {FG}}\mathop \sum \limits_{i.j = 1}^{FG} {{\left( {{p_z}\left( {i.j} \right) - {{\rm \mu}_z}} \right)}^2}}$$

(4) $${\rm Variance}\,\left( {{\rm \sigma}_z^2} \right) = \displaystyle{1 \over {FG}}\mathop \sum \limits_{i,j = 1}^{FG} {\left( {{p_z}\left( {i.j} \right) - {{\rm \mu}_z}} \right)^2}$$

(5) $${\rm RMS}= \sqrt {\mathop \sum \limits_{i.j = 1}^{F.G} {{\left| {{{\rm \mu}_z}\left( {i.j} \right)} \right|}^2}}$$

(6) $${\rm Range}\ ({R_z}) = {p_z}_{\rm max} - \; {p_z}_{\rm min}$$

Where ${p_z}_{\rm max}$ and ${p_z}_{\rm min}$ are the maximal and minimal pixel values in an image block *z*.

###### Discrete Wavelet Transform

**Figure 7:**
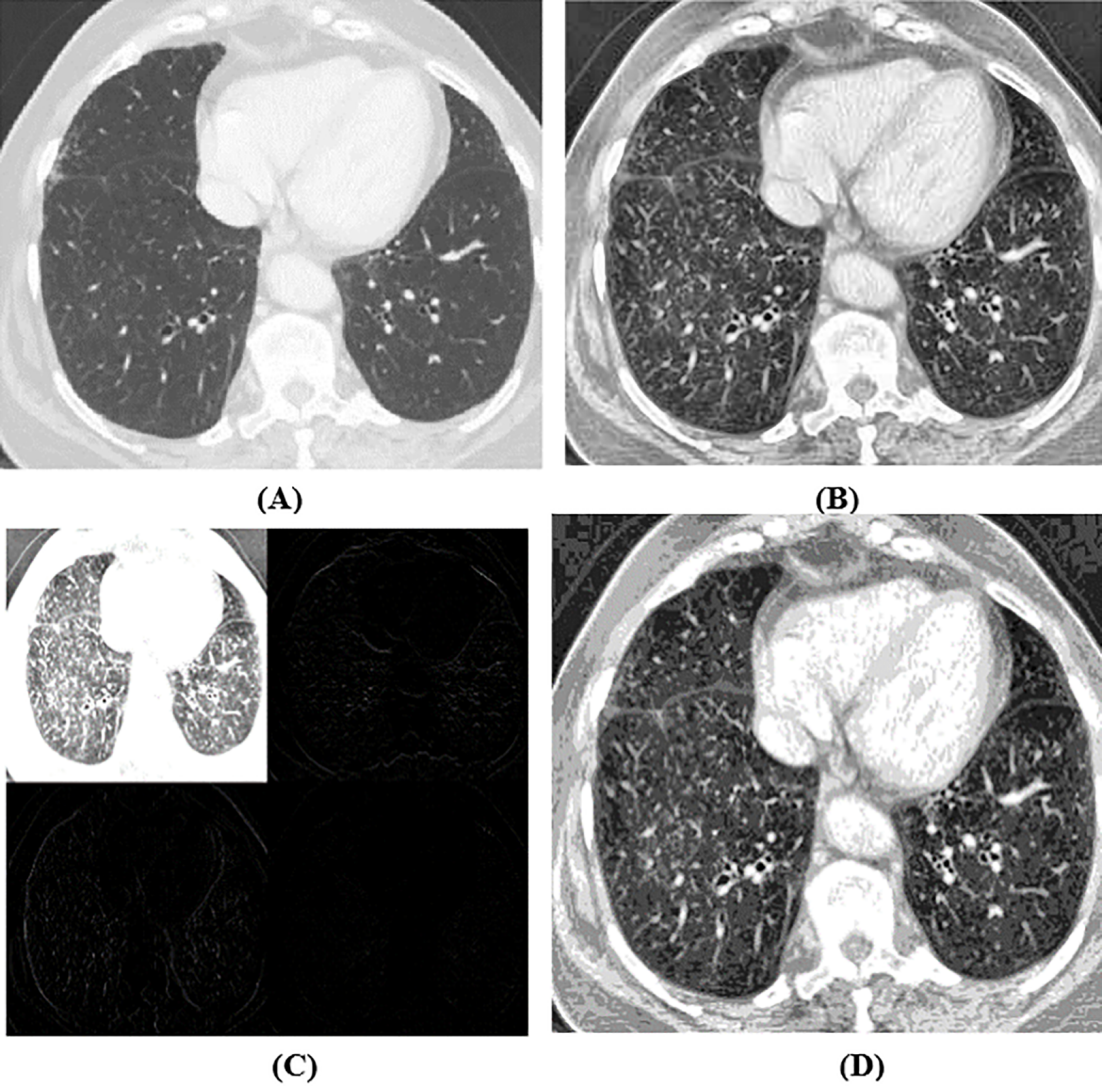
The illustration of the handcrafted features. (A) Original non-COVID-19 sample, (B) enhanced image using CLAHE method, (C) the first level DWT decomposition and (D) GLCM features.

Discrete wavelet transform (DWT) is a common approach to extract features in the medical image processing field (Lahmiri & Boukadoum, 2013; Srivastava & Purwar, 2017). DWT provides time-frequencies demonstration by decomposing an image using a set of orthogonal basis functions (Ortho-normal). DWT has a set of transforms each with a number of wavelet basis functions. In the case of 2-D images, one level 2-D DWT is employed; where a 1D-DWT is applied for every dimension distinctly to attain four sets of coefficients. The four coefficients generated from the 2-D DWT are the approximation coefficients CA_1_, and three detail coefficients CD_1_. The detail coefficients comprise the horizontal, vertical, and diagonal coefficients correspondingly. Multilevel 2-D DWT may be used as well to accomplish multiple decomposing levels. The multilevel decomposition is achieved by convolving the approximation components created in the preceding decomposition level into several high and low-pass filters (Mallat, 1989; Anitha & Murugavalli, 2016). An example of a first level decomposition for a non-COVID-19 sample is illustrated in Fig. 7C.

In the proposed FUSI-CAD system, 4-decomposing levels of DWT are applied to the images. The 4th decomposition level of 2D-DWT usually enhances the performance when used with medical as stated in Chato, Chow & Latifi (2018) and this is proved in the results section. In this paper, Haar wavelet was employed as a wavelet basis function. After performing four-level decompositions, the approximate, horizontal, vertical, and diagonal coefficients of the fourth decomposing stage were extracted to form a feature subset. The number of features extracted from the fourth level of DWT was 196.

###### Grey-level co-occurrence matrix

Grey-level co-occurrence Matrix (GLCM) approach is a common method for pulling out texture features from images (Haralick, Shanmugam & Dinstein, 1973). GLCM method is used to extract surface features such as; contrast, homogeneity, correlation, and energy in the images. GLCM features are determined using Eqs. (7)–(10). The length of the features extracted using GLCM was 64. An example of the GLCM features of a non-COVID-19 sample is shown in Fig. 7D.

(7) $${\rm Contrast} = \mathop \sum \limits_{n = 0}^{G - 1} {n^2}\left\{ {\mathop \sum \limits_i^F \mathop \sum \limits_J^G P\left( {i.j} \right)} \right\}\; .\; \left| {i - j} \right| = n$$

(8) $${\rm Correlation}= \; \mathop \sum \limits_i^F \mathop \sum \limits_j^G \displaystyle{{\left\{ {i \times j} \right\} \times P\left( {i.j} \right) - \left\{ {{{\rm \mu} _x} \times {{\rm \mu} _y}} \right\}} \over {{{\rm \sigma} _x} \times {{\rm \sigma} _y}\; }}$$

(9) $${\rm Energy}= \; \mathop \sum \limits_i^{F - 1} \mathop \sum \limits_j^{G - 1} {\left( {P\left( {i.j} \right)} \right)^2}$$

(10) $${\rm Homogeneity}= \; \; \mathop \sum \limits_i^{F - 1} \mathop \sum \limits_j^{G - 1} \displaystyle{{P\left( {i.j} \right)} \over {1 + \left| {i - j} \right|}}$$

Where *P (i, j)* is a marginal joint probability grey-level co-occurrence matrix.

##### Deep learning feature extraction step

Convolutional neural networks that were pre-trained may be learned from the CT images to either carry out classification or feature extraction tasks. In the feature extraction task, useful deep features were taken out from one of the layers of the CNNs. In this study, instead of using the CNNs as classifiers, DL features were extracted from the “fc7,” the dropout layer named “pool 5 drop 7 × 7 s1,” the “global average pooling 2D layer” (fifth pooling layer), and “node 200” of the fine-tuned AlexNet, GoogleNet, ResNet-18, and ShuffleNet architectures, respectively. The length of DL features extracted from each CNN was 4,096, 1,024, 512, and 544 for AlexNet, GoogleNet, ResNet-18, and SuffleNet respectively.

#### Feature fusion step

The feature fusion step consists of three stages. In the first stage, all DL features extracted from the four CNNs were combined in a concatenated manner. The number of DL features after fusion was 6,176. In the second stage, all handcrafted extracted using DWT, GLCM, and statistical methods were fused. The length of the feature space for the handcrafted features was 772. As mentioned in related image classification problems that combining DL and handcrafted features may enhance the performance of classification problems (Hasan et al., 2020; Wei et al., 2019a; Hansley, Segundo & Sarkar, 2018). Thus, in the third stage, the FUSI-CAD system was constructed by fusing both the DL and HC features. to examine the effect of this fusion on the performance of FUSI-CAD system in distinguishing between COVID-19 and non-COVID-19 cases. The performance of the FUSI-CAD system was compared with the first two stages to test the effect of fusing multiple DL features with three HC features on the performance of the CAD system.

#### Classification step

This step is responsible for classifying CT images to COVID-19 and non-COVID-19 using the SVM classifier. SVM is a machine learning method that uses statistical learning theory to perform classification. SVM classifier is used to construct the optimal hyperplane with the highest margin to separate between two groups of CT datasets. Support vectors represent the data points, which exist on the margin. SVM employs a kernel function to convert the feature space into a new domain to make the separation between the two classes of the datasets easier (Wagh & Vasanth, 2019). In this paper, the cubic kernel function is chosen as it achieved the highest results.

## Performance Evaluation

The performance of the proposed FUSI-CAD system is calculated with several metrics such as accuracy, sensitivity, precision, specificity, F1-score, diagnostic odds ratio (DOR), and area under receiving operating characteristics (AUC). The equations used to calculate such metrics are shown below in Eqs. (11)–(16).

(11) $$\rm {Accuracy} = \displaystyle{{TP + TN} \over {TN + FP + FN + TP}}$$

(12) $$\rm Sensitivity = \displaystyle{{TP} \over {TP + FN}}$$

(13) $$\rm Specificity = \displaystyle{{TN} \over {TN + FP}}$$

(14) $$\rm Precision = \displaystyle{{TP} \over {TP + FP}}$$

(15) $$\rm F1 - Score = \displaystyle{{2 \times TP} \over {\left( {2 \times TP} \right) + FP + FN}}$$

(16) $$\rm DOR = \displaystyle{{TP \times TN} \over {FP \times FN}}$$

Where TP is the sum of COVID-19 images that are properly classified, TN is the sum of non-COVID-19 images that are properly classified. FP is the sum of non-COVID-19 images that are not correctly classified as COVID-19. FN is the sum of COVID-19 images that are not correctly classified as non-COVID-19.

## Experimental Set up

### Parameter setting

Some parameters are adjusted for the pre-trained CNNs. The mini-batch length and validation frequency are 10 and 4 correspondingly the total epochs are equal to 20 and the primary learning rate for the four CNNs is 10^−4^. The L-regularization is 0.0005. However, all other parameters are not altered. These arrangements are to approve that the parameters are modified for classifying medical CT images of COVID-19. Stochastic gradient descent with momentum (SGDM) is applied for the optimization. To verify the performance of the proposed FUSI-CAD system, 5-folds cross-validation is employed.

### Augmentation

Augmentation is an essential process that is usually made to enlarge the size of the dataset, which has a small number of images. This procedure is done as most likely the classification model is trained with an insignificant amount of data over-fit (Ravì et al., 2016). Overfitting means that the model will remember the details of the training set and will not execute well on testing sets. The augmentation method used to generate new lung CT images from the training data in this paper are translation, flipping, scaling, and rotation. Every original CT image was flipped then translated and scaled in x and y-directions with pixel range (−30 to 30) and scale range (0.9–1.1), respectively. Furthermore, the images were rotated with an angle range (0–180) degrees.

## Results

This study proposed a novel CAD system called FUSI-CAD based on the fusion of multiple CNNs and three handcrafted features to classify COVID-19 and non-COVID-19 cases. In this section, the classification results of the three stages of the features fusion are presented.

### Deep features fusion stage

In this stage, the deep features from AlexNet, GoogleNet, ShuffleNet, and ResNet-18, CNNs were extracted, combined, and used to construct a cubic SVM classifier. The performance of the cubic kernel SVM classifier trained with the fused deep features was compared to the one constructed with DL features extracted from the four CNNs individually as shown in Table 3.

**Table 3:** The evaluation metrics for the cubic kernel SVM classifier constructed with the fused DL features compared to SVM classifiers trained with each DL feature.

CNN	Accuracy (std)	AUC (std)	Sensitivity (std)	Specificity (std)	Precision (std)	F1 score (std)	DOR (std)
AlexNet	94.8% (0.001)	0.99 (0)	0.95 (0)	0.948 (0.005)	0.947 (0.005)	0.949 (0.003)	342.001 (30.593)
GoogleNet	96.7% (0.003)	0.99 (0)	0.97 (0)	0.963 (0.004)	0.962 (0.005)	0.966 (0.003)	829.889 (113.608)
ShuffleNet	96.3% (0.001)	0.99 (0)	0.97 (0)	0.961 (0.001)	0.96 (0.001)	0.965 (0.001)	776 (0)
ResNet-18	**97.6%** (0.003)	**1.00** (0)	**0.975** (0.006)	**0.975** (0.006)	**0.975** (0.006)	**0.975** (0.006)	**1,723.223** (714.441)
DL FUSION	**98.6%** (0.001)	**1.00** (0)	**0.981** (0.001)	**0.99** (0)	**0.99** (0)	**0.986** (0)	**4,851** (0)

**Note:**

Bold values indicate the highest results.

For the single deep feature classification, the classification scores for ResNet-18 CNN features achieved the highest scores compared to the other CNN features. This was clear from Table 3, as the accuracy and AUC achieved were 97.6% and 1.00 (100%), respectively. However, the sensitivity, specificity, precision, and F1 score attained to 0.975 (97.5%) each. Moreover, the classification scores for the individual features of the GoogleNet and ShuffleNet CNNs were almost the same. This was because the accuracy achieved was 96.7% and 96.3% for GoogleNet and ShuffleNet CNNs, respectively. In addition, the AUC and the sensitivity were the same for both features achieving 0.99 (99%) and 0.97 (97%), respectively. However, there was a slight change in the scores of the specificity, precision, and F1 score for the two CNNs features. The scores achieved for GoogleNet and ShuffleNet CNNs features were 0.963 (96.3%) and 0.961 (96.1%), 0.962 (96.2%) and 0.96 (96%), and 0.966 (96.6%) and 0.965 (96.5%) for the specificity, precision, and F1 score, respectively. Conversely, the AlexNet CNN individual features achieved the least classification scores achieving 94.8%, 0.99 (99%), 0.95 (95%), 0.948 (94.8%), 0.947 (94.7%) and 0.949 (94.9%), for accuracy, AUC, sensitivity, specificity, precision, and F1 score, respectively.

On the other hand, for the deep feature fusion, it was clear that the fusion had improved the performance metrics of the SVM classifier compared to each DL features. This was because the accuracy, AUC, sensitivity, specificity, precision, and F1 score increased to 98.6%, 1.00 (100%), 0.981 (98.1%), 0.99 (99%), 0.99 (99%), and 0.986 (98.6%), respectively. Furthermore, the DOR had increased to 4851, which was also greater than DORs of the AlexNet, GoogleNet, ShuffleNet, and ResNet-18 respectively.

### Handcrafted Features Fusion Stage

Three types of handcrafted (HC) features were extracted from the CT images; the statistical features, DWT, and GLCM features. As stated previously, eight statistical features; entropy, mean, variance, standard deviation, minimum, maximum, and range were mined from the images. Afterward, these features were fused in one feature vector and classified using the cubic kernel SVM classifier giving an accuracy, AUC, and sensitivity of 97.6%, 0.99 (99%), and 0.971 (97.1%), respectively. Additionally, the specificity and precision accomplished the same score of 0.98 (98%). However, the F1 score and DOR achieved 0.976 (97.6%) and 1584.334, respectively.

**Figure 8:**
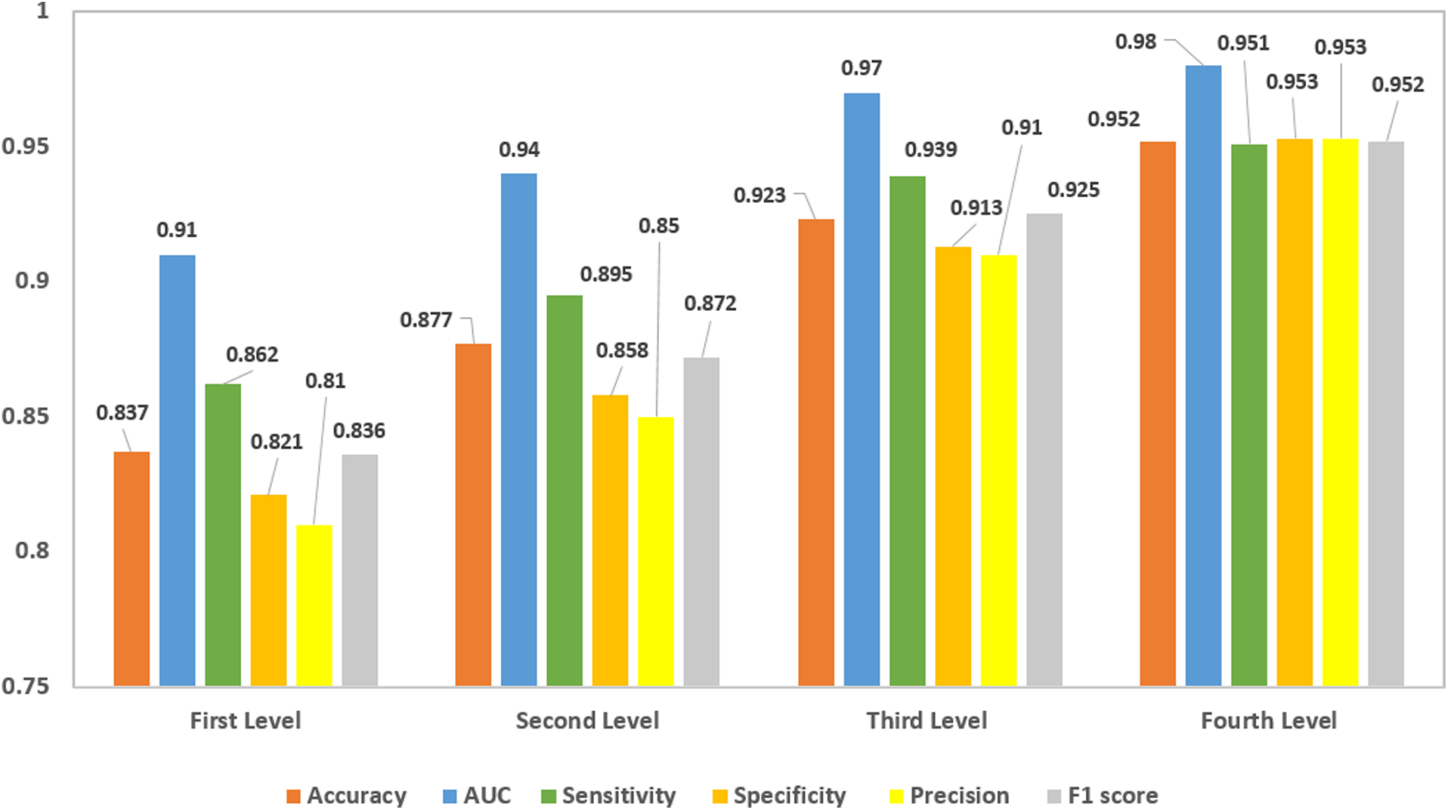
The classification scores for the four levels of DWT decomposition.

Furthermore, for the DWT features, four-level decomposition was performed on the CT images. The classification scores of the four levels of DWT were shown in Fig. 8. The classification scores of the coefficients of the fourth level DWT decomposition attained the highest scores compared to the other three levels, as it was clear in Fig. 8. The accuracy and AUC achieved were 95.2% and 0.98 (98%), respectively.

Moreover, the GLCM features were extracted achieving an accuracy, AUC, sensitivity, specificity, precision, F1 score, DOR of 93.6%, 0.98 (98%), 0.931 (93.1%), 0.94 (94%), 0.94 (94%), 0.936 (93.6%) and 208.143, respectively.

Afterward, the three HC features were fused and classified using a cubic SVM classifier. Since the coefficients of the fourth level DWT achieved the highest scores comparing to the other three DWT levels. Therefore, these features along with the GLCM and statistical features were fused forming one feature vector and used to construct a cubic SVM classifier. This SVM performance was compared with the performance of the SVM classifiers constructed separately with the features extracted for each of the four levels of DWT as well as GLCM features and the statistical features as shown in Table 4.

**Table 4:** The evaluation metrics for the SVM classifier constructed with each individual and fused HC features.

Handcrafted (HC) features	Accuracy (std)	AUC (std)	Sensitivity (std)	Specificity (std)	Precision (std)	F1 score (std)	DOR (std)
Statistical features	97.6% (0.002)	0.99 (0)	0.971 (0.001)	0.98 (0.001)	0.98 (0.001)	0.976 (0.001)	1584.334 (0.001)
Fourth level DWT	95.2% (0.001)	0.98 (0.001)	0.951 (0.001)	0.953 (0.005)	0.953 (0.005)	0.952 (0.003)	389.5 (45.89)
GLCM features	93.6% (0.002)	0.98 (0.001)	0.931 (0.001)	0.94 (0.001)	0.94 (0.001)	0.936 (0.001)	208.143 (0.001)
HC FUSION	**98%** (0.003)	**0.995** (0.006)	**0.976** (0.006)	**0.984** (0.006)	**0.984** (0.006)	**0.98** (0.005)	**2,846.455** (1,607.139)

**Note:**

Bold values indicate the highest results.

The fusing of the three HC features had improved the performance of the SVM classifier, as it was clear in Table 4. The accuracy and AUC increased to 98% and 0.995 (99.5%). Moreover, the sensitivity and specificity were improved as well yielding to 0.976 (97.6%) and 0.984 (98.4%). In addition, the precision and F1 score were raised to 0.984 (98.4%) and 0.98 (98%). Furthermore, the DOR had increased to 2846, which was greater than the DORs of each HC features.

### Deep and handcrafted features fusion stage (FUSI-CAD System)

The FUSI-CAD system proposed the fusion of DL and HC features to examine the effect of this fusion on the performance of COVID-19 diagnosis. Table 5 shows a comparison of DL, HC features fusion, and the proposed FUSI-CAD system (DL and HC features fusion) using a cubic kernel SVM classifier.

**Table 5:** A comparison between the classification scores obtained by the DL, HC feature fusion, and the FUSI-CAD system.

	Accuracy (std)(95% Confidence Interval)	AUC (std) (95% Confidence Interval)	Sensitivity (std) (95% Confidence Interval)	Specificity (std) (95% Confidence Interval)	Precision (std) (95% Confidence Interval)	F1 score (std) (95% Confidence Interval)	DOR (std) (95% Confidence Interval)
DL FUSION	98.6%	1.00	0.981	0.99	0.99	0.986	4,851
	(0.001)	(0)	(0.001)	(0)	(0)	(0)	(0)
	[0.985–0.986]	[1–1]	[0.981–0.981]	[0.99–0.99]	[0.99–0.99]	[0.986–0.986]	[4,851–4,851]
HC FUSION	98%	0.995	0.976	0.984	0.984	0.98	2,846.455
	(0.003)	(0.006)	(0.006)	(0.006)	(0.006)	(0.005)	(1,607.139)
	[0.978–0.981]	[0.992–0.997]	[0.973–0.978]	[0.981–0.986]	[0.981–0.986]	[0.977–0.983]	[1,968.2–3,724.7]
FUSI-CAD	**99%**	**1.00**	**0.99**	**0.99**	**0.99**	**0.99**	**9,801**
	**(0.002)**	**(0)**	**(0)**	**(0)**	**(0)**	**(0)**	**(0)**
	**[0.988–0.991]**	**[1–1]**	**[0.99–0.99]**	**[0.99–0.99]**	**[0.99–0.99]**	**[0.99–0.99]**	**[9,801–9,801]**

**Note:**

Bold values indicate the highest results.

**Figure 9:**
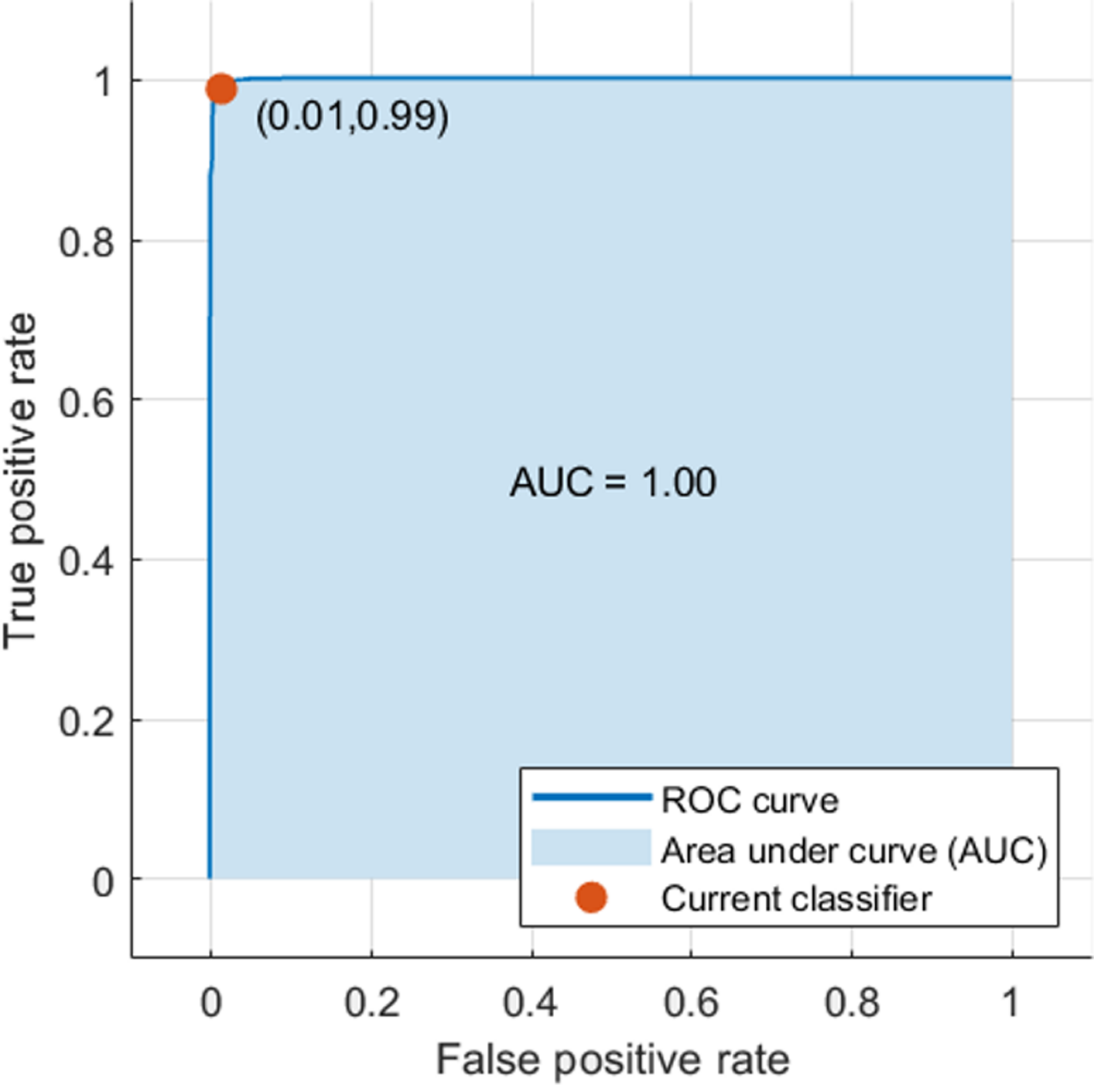
The ROC curve for the FUSI-CAD system.

All the classification results achieved by the FUSI-CAD system increased, as it was obvious in Table 5. The accuracy, sensitivity, specificity, precision, and F1 score increased to 99% each. In addition, the AUC rose to 1.00 (100%), as shown in the computed ROC curve in Fig. 9.

**Figure 10:**
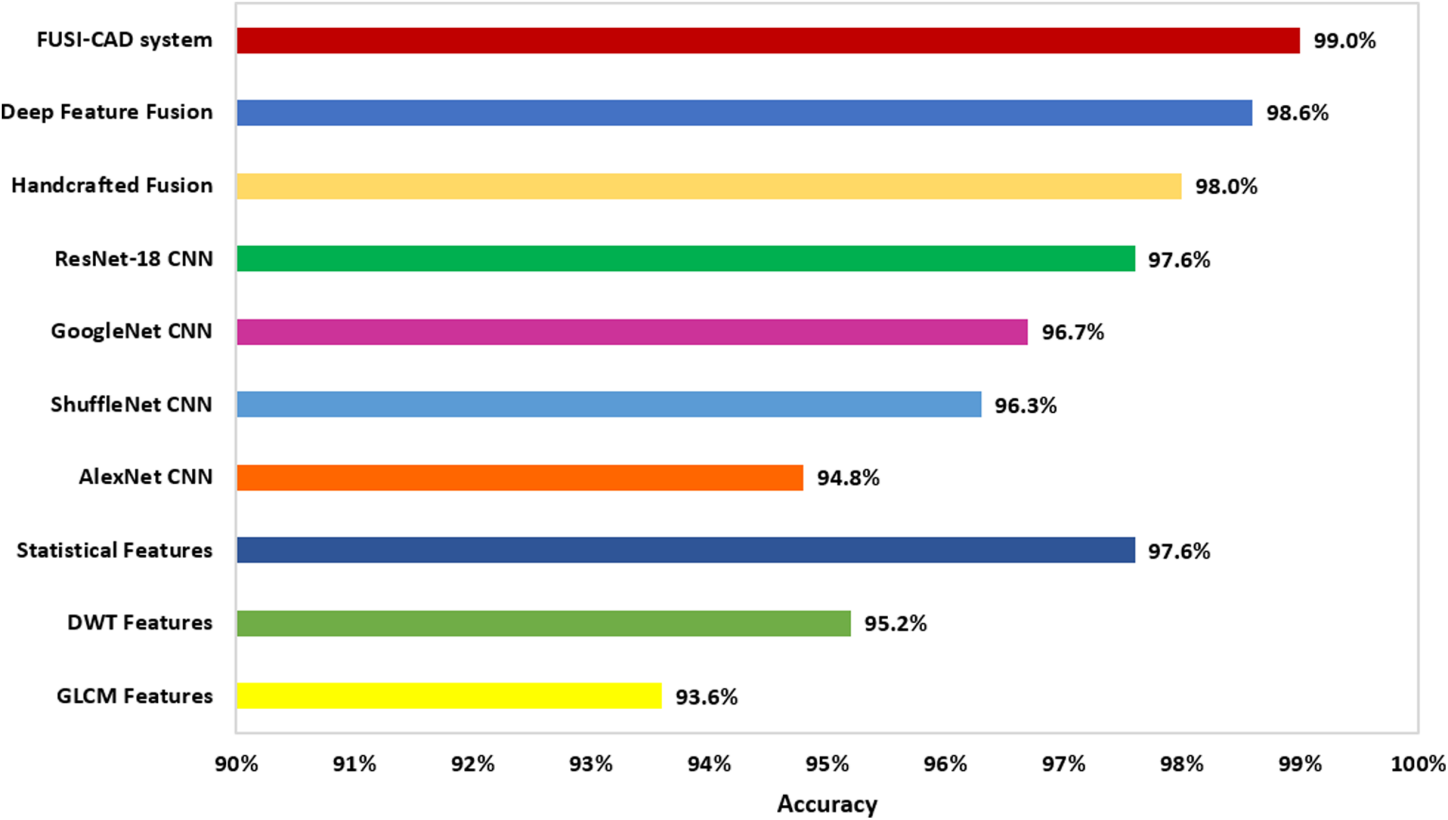
Comparisons of individual DL features, individual handcrafted features, DL feature fusion, handcrafted feature fusion and the proposed FUSI-CAD system.

A comparison between the accuracies of SVM classifiers constructed using individual DL features, individual HC features, DL feature fusion, HC feature fusion, and the proposed FUSI-CAD system is given in Fig. 10. As shown in Fig. 10 the accuracy of the proposed FUSI-CAD system attained the highest score.

## Discussions

Currently, the novel coronavirus is considered one of the global terrific health crisis. A quick and accurate diagnostic tool is vital to manage the propagation of this pandemic disease and assist radiologists in the diagnosis especially in high load conditions. The RT-PCR laboratory test is a well-known method to diagnose COVID-19, but it has poor sensitivity and inadequate availability. The test is also expensive, time-consuming, and requires a well-equipped laboratory for analysis (which is the main challenge particularly in developing countries). Therefore, a faster and efficient alternative diagnostic tool is of crucial need to help the front-line professionals to attain a fast and precise diagnosis.

Medical imaging techniques such as CT is an effective tool to visualize the lungs and can assist the early diagnosis of COVID-19. However, it cannot achieve efficient diagnosis when used alone because of the likeness between patterns of the novel coronavirus and other types of pneumonia, which could confuse specialists and lead to misdiagnosis. On the other hand, CAD systems based on AI techniques have proven to have stronger discriminating ability to distinguish COVID-19 and non-COVID-19 patients, and more accurate and faster capabilities in the diagnosis the COVID-19 compared to the pathogenic exam, which consequently lessens the time desired for disease control (Butt et al., 2020; Shi, Han & Zheng, 2020).

In this study, a faster and more accurate resolution was proposed instead of the RT-PCR test. The proposed resolution presented a novel CAD system called FUSI-CAD system. Recent studies based on related image classification problems have suggested that fusing DL and handcrafted features can improve the performance of the classification model (Hasan et al., 2020; Wei et al., 2019a; Hansley, Segundo & Sarkar, 2018). Therefore, the FUSI-CAD system was based on the fusion of multiple DL features and handcrafted features extracted from CT images to investigate the effect of this fusion on the performance of the CAD system. Moreover, the proposed CAD system examined the influence of fusing multiple DL features extracted from four pre-trained CNNs on the classification performance as in stage (1). Furthermore, it studied the impact of fusing three handcrafted features such as DWT, GLCM, and statistical features on the classification performance as well as illustrated in stage (2). The proposed system is considered an important trial involving a simple to set up, low cost, and automated CAD system that can attain an accurate, effective, and fast diagnostic tool. Throughout this tough period of the global pandemic, the proposed FUSI-CAD system has huge potential to be considered as a COVID-19 diagnostic tool. It can attain early diagnosis of the novel corona disease, thus averting its rapid propagation.

The FUSI-CAD system is an AI technique, which has a more powerful ability to distinguish between COVID-19 and non-COVID-19 CT images than manual diagnosis (Butt et al., 2020). The superiority of AI techniques were confirmed in related studies by various authors (Bai et al., 2020; Chen et al., 2020; Ardakani et al., 2020), who compared the accuracy of their CAD systems based on AI techniques, with the accuracy achieved by a manual diagnosis of radiologists without the aid of a CAD system. The performance attained by these studies verified that the AI based CAD systems were greater than the manual diagnosis by radiologists. The authors in Bai et al. (2020) indicated that their CAD system based on AI reached higher test accuracy (96% vs. 85%), the sensitivity of (95% vs. 79%), and specificity (96% vs. 88%) than radiologists. Similarly, in Ardakani et al. (2020) the results attained by the radiologist were worse than the authors’ proposed AI-based CAD system, with a sensitivity of (89.21% vs. 98.04%), a specificity of (83.33% vs. 98.04%), and an accuracy of (86.27% vs. 99.5%). Whereas, in Chen et al. (2020), the authors indicated that their AI based CAD system is capable of lowering the manual radiologist’s diagnosis time by 65%.

This study proved that DL feature fusion could enhance the diagnosis of COVID-19 as the accuracy of the SVM classifiers had increased to 98.6%, which was higher than that of the individual DL features extracted from AlexNet, GoogleNet, ShuffleNet, and ResNet-18 CNNs as shown in Fig. 10. Moreover, the study indicated that fusing the handcrafted features had improved the accuracy of the SVM classifier to reach 98%, which was better than that of the DWT, GLCM, and statistical features separately. Furthermore, the performance of the proposed FUSI-CAD system verified that fusing DL and HC features had successfully improved the accuracy to reach 99%, which was higher than the other individual features of the DL methods, HC methods, the fused DL features, and fused HC features as shown in Fig. 10.

The FUSI-CAD system performance was compared with recent related studies based on the same dataset to verify its competence. Table 6 shows a comparison between the performance metrics of the proposed FUSI-CAD system and recent related studies based on the SARS-CoV-2 CT-scan dataset. The accuracy (99%), AUC (1.00), sensitivity (99%), specificity (99%), precision (99%), and F1 score (99%) were higher than the other methods. Soares et al. (2020) employed different separate CNNs such as; x-DNN, ResNet, GoogleNet, VGG-16, and AlexNet to classify COVID-19. They also used AdaBoosting and decision trees (DT) classifiers, however DT performance was lower than CNN. The highest result was achieved by x-DNN reaching an accuracy of 97.38%, AUC of 97.36%, sensitivity of 95.53%, precision of 99%, and F1 score of 97.31%. Whereas, the authors of Panwar et al. (2020) presented a deep transfer learning algorithm that quickens the detection of COVID-19. This method used gradient weighted class activation mapping (Grad-CAM) to visualize and plot class activation maps. This can assist in explaining more information about the CNN while carrying out the detection process. An accuracy of 95%, sensitivity of 96%, precision of 95%, and F1 score of 95% were attained, which were lower than FUSI-CAD system because this method used only one type of CNN to perform the detection. On the other hand, Pathak, Shukla & Arya, (2020) proposed a deep bidirectional long short-term memory (LSTM) network with mixture density network (DBM) model. A memetic adaptive differential evolution (MADE) algorithm was employed to tune the hyper-parameters of the DBM model. The model achieved an accuracy of 98.37%, AUC of 98.2%, sensitivity of 98.87%, precision of 98.74%, and F1 score of 98.14%. As it can be noted from Table 6 that the authors of Soares et al. (2020), Panwar et al. (2020) and Pathak, Shukla & Arya (2020) have utilized different individual CNNs networks. These authors did not neither fuse several CNNs architectures nor combine DL features with handcrafted features. However, the FUSI-CAD system fused multiple DL features with three HC features, which improved the performance of the CAD system and this is considered the main advantage of the FUSI-CAD system.

**Table 6:** Comparison between the proposed FUSI-CAD system and the recent related studies on the SARS-CoV-2 CT-scan dataset.

Paper	Method	Accuracy (%)	AUC (%)	Sensitivity (%)	Precision (%)	F1 score (%)
Soares et al. (2020)	x-DNN	97.38	97.36	95.53	99	97.31
	ResNet	94.96	94.98	97.15	93	95.03
	GoogleNet	91.73	91.79	93.5	90.2	91.82
	VGG-16	94.96	94.96	95.43	94.02	94.97
	AlexNet	93.75	93.68	92.28	94.48	93.61
	Decision Tree	79.44	79.51	83.13	76.81	79.84
	Adaboost	95.16	95.19	96.71	93.63	95.14
Panwar et al. (2020)	CNN	95	–	96	95	95
Pathak, Shukla & Arya (2020)	LSTM*	98.37	98.2	98.87	98.74	98.14
Proposed FUSI-CAD system		**99**	**100**	**99**	**99**	**99**

**Notes:**

* LSTM: Long short-term memory.

Bold values indicate the highest results.

These results proved the competitiveness of the FUSI-CAD system compared to other studies. Moreover, these results confirmed that the proposed FUSI-CAD system was capable of overcoming the constraint of using CT images only to diagnose the COVID-19 disease. Furthermore, according to what had been stated in Colquhoun (2014), Ellis (2010), Attallah (2020) that for a CAD system to be reliable, it must attain a sensitivity larger than or equal to 80%, specificity more than or equal to 95%, precision higher than or equal 95%, and a DOR greater than or equal 100. Therefore, the performance of the proposed FUSI-CAD system as in Table 5 verified that it is reliable and can be used to diagnose COVID-19. This was because the sensitivity, specificity, and precision achieved 99%, in addition to the DOR was 9802. The competing performance of the FUSI-CAD system revealed the idea of producing a diagnostic software tool by IT (information technology) solution companies. This tool can be portable and desirable to the end-user, such as radiologists or specialists, to assist the diagnosis procedure of the COVID-19.

The performance of the proposed FUSI-CAD system was also compared with the related work presented in Table 1; it was observed that the CNN constructions were different from those used in the FUSI-CAD system. Concerning the ResNet CNN constructions, it was clear that the authors of the related work employed it with several layer architectures as in Butt et al. (2020), Li et al. (2020), Jin et al. (2020a, 2020b), Song et al. (2020b) and Ardakani et al. (2020). Butt et al. (2020) utilized the ResNet-18 and ResNet-23 CNNs to identify COVID-19 samples attaining an accuracy of 86.7%, which was beneath that of the FUSI-CAD system. This was because they used each network individually to perform the classification. Alternatively, Jin et al. (2020a) did the classification process using ResNet-152 CNN achieving an accuracy of 94.8%, which was lower than the accuracy of the FUSI-CAD system built with a fewer number of images. Li et al. (2020), Song et al. (2020b) and Jin et al. (2020b) employed the ResNet-50 CNN reaching an accuracy of 89.5%, 86%, and 94.8% respectively. The accuracies achieved by Li et al. (2020) and Song et al. (2020b), were much lower than the proposed FUSI-CAD system. This was because they used individual ResNet-50 networks trained with small amount of input images. However, the accuracy obtained by Jin et al. (2020b) was higher than Li et al. (2020) and Song et al. (2020b). This was due to the large amount of data employed by Jin et al. (2020b) method compared to Li et al. (2020) and Song et al. (2020b), but it was still lower than FUSI-CAD system. The reason for that was that FUSI-CAD system combined several DL features from CNNs architectures with three handcrafted textural analysis features. Instead, Ardakani et al. (2020) employed ResNet-101 CNN and achieved an accuracy of 99.51%. The reason for this high accuracy was that the authors used very high-resolution images and they divided the images into patches. Amyar, Modzelewski & Ruan (2020) and Chen et al. (2020) used the U-Net for the segmentation and/or classification procedures, the accuracies achieved were 86%, and 95.2%, respectively. The high accuracy achieved by Chen et al. (2020) was due to the huge number of images used to train their U-Nets, but it was still lower than the accuracy of proposed FUSI-CAD system. This was because FUSI-CAD system applied the feature fusion process, which enhanced the performance. Zheng et al. (2020) used a U-Net for segmenting CT images, and then they built a new CNN with eight layers. The accuracy attained 90.9%, which was much lower than achieved in FUSI-CAD system.

Furthermore, He et al. (2020) had constructed a CAD system for coronavirus diagnosis based on DenseNet-169 CNN attaining an accuracy of 86%. Although, the DenseNet network architecture can perform well in case trained with a huge number of images, which was not the case in He et al. method. On the other hand, the authors of Hasan et al. (2020) utilized DL features extracted from a new CNN architecture with a handcrafted feature extracted method and attained an accuracy of 99.68%, which was slightly higher than that that of the FUSI-CAD system. The reason for the slightly outperformance of Hasan et al. (2020) method was that the authors constructed a CNN with few number of convolutional layers to decrease the over-fitting by reducing the CNN construction complexity. Conversely, Fang et al. (2020) used the handcrafted features only and discarded the advantages of DL techniques and therefore, they attained a low AUC of 0.826 (82.6%).

To test and validate the statistical significance of the results obtained, a one-way analysis of variance (ANOVA) test was performed by the repeated fivefold cross-validation procedure. The null hypothesis Ho for all classification was that the mean accuracies achieved in each experiment. Table 7 shows the results of ANOVA test made for the fused deep features (stage 1). Table 8 shows the results of ANOVA test made for the fused handcrafted features (stage 2). Table 9 presents the results of ANOVA test executed for the fused the fusion of the multiple deep features and handcrafted features (FUSI-CAD). It can be observed from Tables 7–9, that the *p*-values achieved were lower than 0.05. Therefore, it can be concluded that there was a statistically significant difference between the results calculated for each stage. Also the results of the test verify that FUSI-CAD is reliable. Moreover, the 95% confidence interval calculated for each stage of the proposed system proves that FUSI-CAD is reliable.

**Table 7:** The ANOVA test details for the deep feature fusion.

Source of variation	SS	df	MS	*F*	*p*-Value
Columns	0.00209	5	0.00042	9384	<0.005
Error	0	54	0		
Total	0.00209	59			

**Table 8:** The ANOVA test details for the handcrafted feature fusion.

Source of variation	SS	df	MS	*F*	*p*-Value
Columns	0.00234	5	0.00047	20.56	<0.005
Error	0.00137	60	0.00002		
Total	0.00371	65			

**Table 9:** The ANOVA test details for FUSI-CAD system.

Source of variation	SS	df	MS	*F*	*p*-Value
Columns	0.00087	5	0.00017	728.44	<0.005
Error	0.00001	54	0		
Total	0.00088	59			

Although the FUSI-CAD system outstanding performance, it still has some limitations. Meanwhile, the proposed system can only differentiate between COVID-19 and non-COVID-19 images, but it is crucial to discriminate COVID-19 cases from other categories of pneumonia as well. In addition, the performance of the FUSI-CAD system was not compared with a manual diagnosis by a radiologist. Future work will focus on extending the model so that it would be able to identify other types of pneumonia, employing imaging techniques such as X-rays and constructing other types of CNNs.

## Conclusions

Numerous studies have proved that AI techniques are capable of assisting radiologists in accurately identifying the novel coronavirus as well as speeding up the diagnosis process. This paper proposed an accurate and fast diagnostic tool called the FUSI-CAD system for distinguishing COVID-19 from non-COVID-19 cases as an alternative to the laboratory test, which has several drawbacks. FUSI-CAD system is based on the fusion of four DL features with three types of handcrafted features. The results showed that fusing multiple DL features had increased the performance of the classification model compared to the performance of the model constructed with individual DL features. In addition, the outcomes of this study proved that combining the three handcrafted features had increased the accuracy of the diagnosis of COVID-19. Additionally, the FUSI-CAD system had proved that fusing multiple DL and handcrafted features had a positive impact on the diagnosis of COVID-19. Furthermore, it revealed its competitive performance compared to similar studies based on the same dataset. Consequently, the FUSI-CAD system can be successfully used by radiologists to expedite the diagnosis procedure of COVID-19. Additionally, the FUSI-CAD system could help in controlling the present epidemic, accelerate the diagnosis of such virus, deaccelerate its spread, and enable clinicians to enhance the quality of patient management, even in unusual workload circumstances. The FUSI-CAD system must experience additional field-testing afore radiologists can engage it. Moreover, it will probably have to endure regulatory approval by health authorities before its implementation into hospitals.

## Supplemental Information

10.7717/peerj-cs.306/supp-1 Supplemental Information 1 MATLAB code.

## Additional Information and Declarations

### Data Availability

The following information was supplied regarding data availability: The dataset is available at Kaggle: Eduardo Soares and Plamen Angelov, “SARS-COV-2 Ct-Scan Dataset.” Kaggle, DOI 10.34740/KAGGLE/DSV/1199870. The data was described at: A Fully Automated Deep Learning-based Network For Detecting COVID-19 from a New And Large Lung CT Scan Dataset, Mohammad Rahimzadeh, Abolfazl Attar, Seyed Mohammad Sakhaei. medRxiv 2020.06.08.20121541; DOI 10.1101/2020.06.08.20121541.
